# Education of Occupational Therapists in Mental Health: A Global Survey of Educators Regarding Perceived Facilitators and Barriers

**DOI:** 10.3390/ijerph22071009

**Published:** 2025-06-26

**Authors:** Tiago S. Jesus, Pedro C. Monteiro, Ritchard Ledgerd, Claudia von Zweck

**Affiliations:** 1 Division of Occupational Therapy, School of Health & Rehabilitation Sciences, College of Medicine, The Ohio State University Wexner Medical Center, Columbus, OH 43210, USA; 10190509@ess.ipp.pt; 2 School of Health, Polytechnic Institute of Porto, 4200-072 Porto, Portugal; 3 World Federation of Occupational Therapists, 1211 Geneva, Switzerlandcvonzweck@wfot.org (C.v.Z.)

**Keywords:** occupational therapy, occupational therapists, mental health, education, survey

## Abstract

Background: Occupational therapists can address worldwide mental health (MH) needs and workforce shortages. Ways to advance occupational therapy education to build occupational therapist workforce capacity in MH require further investigation. Objective: This study aimed to identify perceived barriers to and facilitators for advancing MH occupational therapy education, as rated by occupational therapy educators from across the world, stratified into groups of high-income countries (HICs) and low- and middle-income countries (LMICs). Method: Global survey, Likert-type, created and distributed by the World Federation of Occupational Therapists. Data were subject to a secondary weighted and subgroup analysis. Results: A total of 155 responses were obtained from occupational therapy educators from 45 countries or territories; 69% of the respondents were from HICs. The weighted analysis showed that educational standards and student interest were large facilitators for both HICs and LMICs. Faculty expertise stood out as a facilitator and the lack thereof as a barrier, both across HICs and LMICs. For HICs, regulation issues, lack of recognition, lack of supervised/fieldwork practice, and lack of workforce demand were frequently reported barriers, whereas lack of teaching resources and practice evidence were often perceived as barriers in LMICs. Conclusions: Capacity building approaches are required to advance MH occupational therapy education, with tailored approaches for HICs and LMICs.

## 1. Introduction

Data from the Global Burden of Disease Study show that MH disorders continue to be one of the leading causes of illness worldwide [1]. In turn, the World Health Organization’s mental health report of 2022 emphasizes that over one billion people worldwide live with an MH condition [2]. People with MH conditions often experience disruptions in their daily life, routines, and occupations [3]. MH involves not merely the absence of mental illness, but also includes social functioning, positive psychological attributes, and the ability to cope with or adapt to challenges of life [3,4].

Occupational therapists are health practitioners who promote occupational performance and engagement in meaningful life activities for individuals and communities that experience, or are at risk of experiencing, an MH, physical, or cognitive condition, impairment, or disability [5]. Occupational therapists have a long history of working in MH contexts [6,7,8]. Occupational therapists promote positive MH and facilitate recovery from MH conditions, allowing individuals and communities to (re-)engage in meaningful life activities such as education, leisure, and work [9,10]. Evidence-wise, a recent systematic review of the effectiveness of occupational therapy for people with a diagnosis of depression found strong evidence for the effectiveness of occupational therapy return-to-work interventions, which is important given the costs associated with mental ill-health and work absence [11]. Another systematic review, with meta-analysis, on clinical effectiveness also found evidence for improvements in occupational performance and well-being as a result of occupational therapy interventions in a wider range of MH conditions [12]. Finally, detailed practice guidelines with associated evidence have been launched on effective practices of occupational therapy for adults living with serious MH conditions [13].

Despite the relevance of occupational therapy, occupational therapists are under-represented in MH practice in many countries [14]. Biennial data collection by the World Federation of Occupational Therapists (WFOT) indicates that MH has been consistently identified as the practice area with the highest vacancy rates for occupational therapists over the 20-year history that the WFOT has monitored the occupational therapy workforce [15]. The number of occupational therapists within the mental health workforce is clearly insufficient to meet health system demands or population needs [14,16].

Underrepresentation of occupational therapy in MH contexts occurs in countries at all income levels, including those with more occupational therapy resources [14,17,18,19]. For instance, in the USA, recent data indicate only 2.2% of the occupational therapist workforce is deployed in MH practice and represents 0.2% of the non-physician MH workforce [17,18]. This low level of involvement occurs within an environment of widely documented shortages of MH professionals in the USA [20].

Advancing the supply and practice of occupational therapists in MH can contribute to reducing worldwide shortages in the MH workforce [10,21,22,23]. In a recent global survey of 1102 occupational therapists working in the MH field, “education/preparation for MH practice” was identified as a facilitator for occupational therapy MH practice [24]. This finding suggests the perspectives of occupational therapy educators would be helpful regarding factors that affect the education and practice of occupational therapists in MH. For example, factors that may affect the education of occupational therapists in MH can relate to the need for enhancing fieldwork opportunities, which can improve students’ interest and competence in MH practice but is often limited in accessibility and scope [25]. Furthermore, having faculty members with clinical experience in MH is also deemed essential to improve the quality of the education of occupational therapists in this field, but these faculty members can be in short supply [21]. While some of the factors have been suggested in the literature, there is no global survey of MH occupational therapy educators on which factors might facilitate or hinder the education and practice of occupational therapists in MH worldwide.

WFOT advances global health and wellbeing through the development, use, and practice of occupational therapy, with a vision to ensure the profession is accessible to all. WFOT engaged in a multistage research process to launch the *Global Strategy for the Occupational Therapy Workforce* [26,27] to guide collaborative work among individuals and organizations towards this vision. The *Global Strategy* emphasizes the need for occupational therapists to be equitably distributed across practice areas, including in MH [27]. The WFOT position statement regarding human resources also reinforces the need for the worldwide strengthening of the MH occupational therapy workforce [22].

Within this context, WFOT conducted a survey to explore factors that influence the education and preparation of occupational therapists for MH practice. In particular, this study aimed to identify perceived barriers and facilitators for advancing MH occupational therapy education, as rated by occupational therapy educators from across the world, stratified into groups of high-income countries (HICs) and low- and middle-income countries (LMICs).

## 2. Method

### 2.1. Overview

The study involved a Likert-type global survey, created and distributed by WFOT through worldwide channels, including circulation by member organizations for wider, snowballing outreach. The survey targeted occupational therapy educators. Participants provided consent for the analysis and public use of their deidentified survey responses.

Data were subject to a secondary weighted and subgroup analysis. A data-sharing agreement was established between WFOT and the Ohio State University (ID: A2024-2516) for this analysis. After de-identification by WFOT, survey responses were shared for secondary analysis.

### 2.2. Survey Design

The survey was designed by WFOT, based on knowledge of international occupational therapy education and relevant research literature, which was identified in a large WFOT scoping review of occupational therapy workforce research [14,26,28] and in other relevant occupational therapy MH research and literature [25,29,30,31,32,33,34]. The WFOT used these sources to draft and refine the survey questions in collaboration with an external researcher.

Table 1 describes the factors rated in the survey regarding their perceived positive (facilitator) or negative (barrier) impact for the education of occupational therapists in MH. A five-point Likert-based scale was used for rating the factors, supplemented by “unknown” or “not applicable” options. The scale defined both the direction (positive/negative) and magnitude (high/low) of the responses and therefore allowed for a weighted analysis. While there are comparative advantages and disadvantages for five- or seven-point Likert-based scales, here, we particularly appreciated the simplicity, intuitiveness, and clear direction of the “low” and “high” positive or negative impact (i.e., four of the grades; two for each side) in addition to the clear neutral stance.

Initially developed in English, the survey was also translated to be disseminated in French, German, and Spanish. The translation process used by WFOT to translate official documents was used, a multi-step process involving use of online software to create machine-translated text that was subsequently checked and double-checked by volunteer translators who frequently perform these roles for WFOT’s documents. The survey text was then loaded onto the SurveyMonkey platform for circulation to the occupational therapists via WFOT member organizations, the WFOT e-newsletter, and other social media platforms.

To respond to the survey, respondents selected their choice of language, consented to participation, provided basic socio-demographic data, and indicated their role in occupational therapy in practice, research, or education. Only respondents in educational roles were presented with the questions in Table 1, whose results are analyzed in this paper. Those who identified as having practice roles answered an alternate set of questions; findings from these study questions are available elsewhere [24].

### 2.3. Survey Procedures

Using the SurveyMonkey platform, WFOT circulated the survey link to the global occupational therapy community in November 2023. Notably, the survey link was sent to over 100 WFOT national member organizations, who were asked to circulate the survey information to their individual members. WFOT also used the WFOT e-newsletter (with over 14,100 recipients), as well as social media for the survey link dissemination. Finally, the survey link was available on the WFOT website. The survey remained open for 6 weeks, with survey reminders included in WFOT e-newsletters twice during this period.

### 2.4. Analysis

The researchers used descriptive statistics and developed all the calculations and graphics of the Likert-based responses for each of the nine survey items using the Excel software (Microsoft Corporation, Redmond, WA, USA). The weighted analysis involved assigning positive and negative weights to the responses: two points to “high positive impact”; one to “low positive impact”; zero to “no impact”; one negative point to “low negative impact”; and two negative points to “high negative impact”. Stacked bar charts were selected to represent the weighted results for each rated factor on a continuum between −100% (for maximum barrier) and +100% (for maximum facilitator) for easier interpretation of the direction and magnitude of the impact. Separate stacked charts of the weighted results were developed for groups stratified by country income level (HIC vs. LMICs, defined by the World Bank), as their contexts might substantially vary.

## 3. Results

### 3.1. Participants

A total of 155 survey responses were obtained from occupational therapy educators, with 69.0% of the respondents (*n* = 107) from HICs. All participants reported at least some experience in educating occupational therapists for working with people with MH needs. Most participants (83%) had >10 years of experience in practice or education, with the current performed role being an educator. Using World Bank regional classifications, the highest number of respondents came from Europe and Central Asia (32%); however, responses came from 45 different countries or territories of seven world regions.

### 3.2. Results for All Respondents

**Figure 1:**
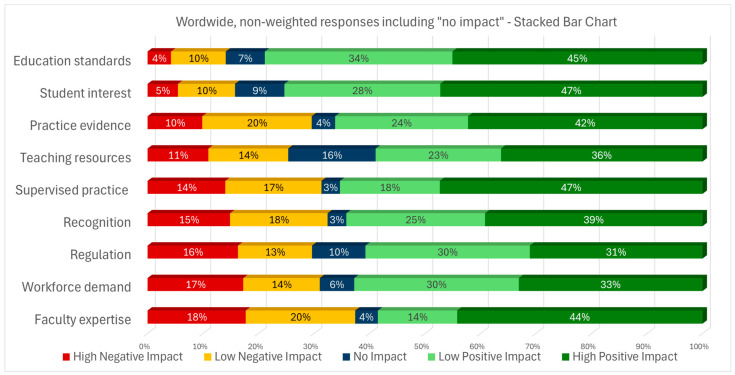
Non-weighted, worldwide results, including “no impact” answers, for the factors positively or negatively impacting the education of occupational therapists for practice in mental health.

Figure 1 provides the non-weighted survey results for all respondents for each of the nine surveyed factors. The factors are ordered from the smallest to largest value for “high negative impact”. *Education standards* and *student interest* had small values for “high negative impact” and large values for “high positive impact”. Among all factors, *faculty expertise* had the largest perceived negative impact at the combined high and low level. Interestingly, this factor also had large values for “high positive impact”.

**Figure 2:**
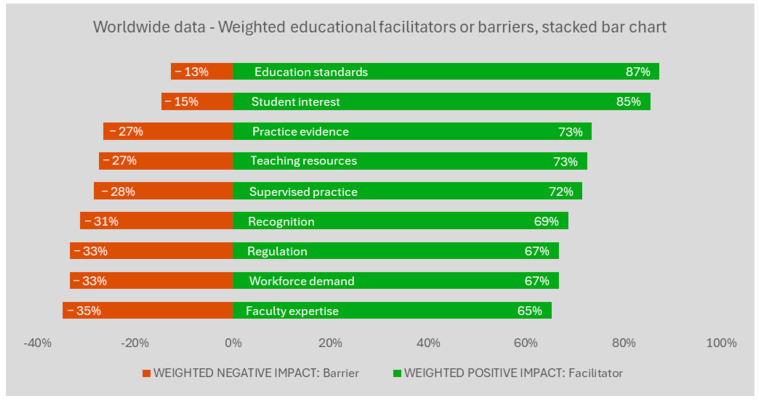
Worldwide results for the weighted barriers or facilitators for the education of occupational therapists in mental health. Legend: Factors were weighted, ranked, and then translated into 100% stacked bars. The values reflect a differential weight for the magnitude of the response direction (i.e., high negative impact answers count double the value of ‘low negative’ responses for the red bar; ‘high positive’ impact answers count double the value of ‘low positive’ responses for the green bar). The weighted results were then ranked, from the most positive to the most negative influence. The weighted, ranked results were translated into 100%-stack bars (i.e., sum of the red and green bar for the same item = 100%). The full description is available on Table 1.

Figure 2 shows the results for the weighted findings as transformed into a two-sided stacked bar chart, after removing the “no impact” answers and assigning a weight for the magnitude. With the data transformation, “high impact” scores have double the value of “low impact” answers in either the positive or negative direction. Figure 2 indicates the results remain similar, with the ranked order of factors the same as in the previous figure.

### 3.3. Stratification by HICs and LMICs

**Figure 3:**
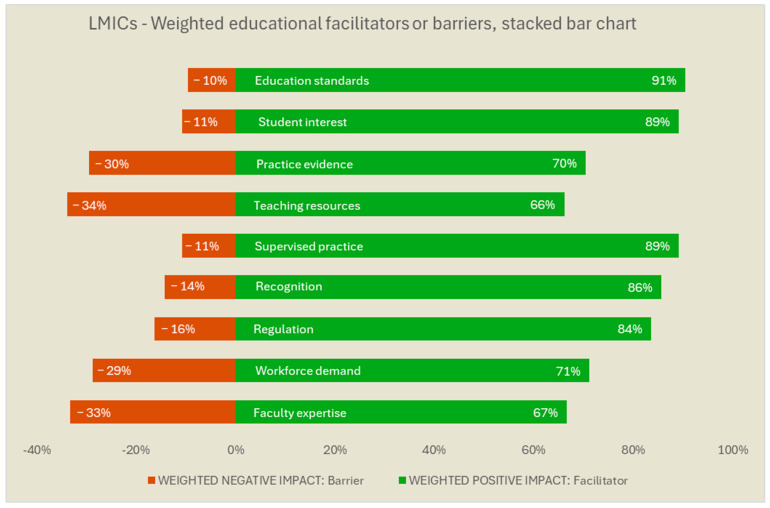
Low and Middle-Income Countries’ (LMICs) weighted barriers and facilitators for the education of occupational therapists in mental health. Legend: The weighted values reflect a differential weight for the magnitude of the response direction (i.e., high negative impact answers count double the value of ‘low negative’ responses for the red bar; ‘high positive’ impact answers count double the value of ‘low positive’ responses for the green bar). The weighted results were ranked in the same order of the non-stratified results, for comparative purposes among countries of varied income levels. The weighted results were translated into 100%-stack bars (i.e., sum of the red and green bar for the same item = 100%) for easier interpretation. The full description is available in Table 1.

**Figure 4:**
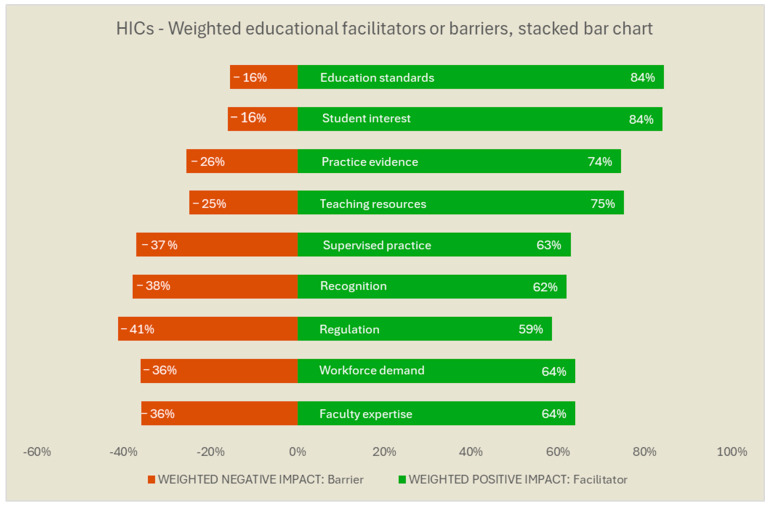
High-Income Countries’ (HICs) weighted barriers and facilitators for the education of occupational therapists in mental health. Legend: The weighted values reflect a differential weight for the magnitude of the response direction (i.e., high negative impact answers count double the value of ‘low negative’ responses for the red bar; ‘high positive’ impact answers count double the value of ‘low positive’ responses for the green bar). The weighted results were ranked in the same order of the non-stratified results, for comparative purposes among countries of varied income levels. The weighted results were translated into 100%-stack bars (i.e., sum of the red and green bar for the same item = 100%) for easier interpretation. The full description is available in Table 1.

Figure 3 and Figure 4 provide the results for the respondents from LMICs and HICs, respectively. In the figures, we used the rank order of the non-stratified group (Figure 2) to facilitate score comparisons. For LMICs, *teaching resources* and *practice evidence* were appraised more often as barriers, in addition to *faculty expertise*. In turn, for HICs, issues such as *regulation*, lack of *recognition*, of *supervised practice*, of *workforce demand*, and of *faculty expertise* were the factors more often reported as barriers. *Education standards* such as accreditation processes *and student interest* in working with people with MH needs were largely rated as facilitators, both for HICs and LMICs.

## 4. Discussion

Occupational therapists can address mental health (MH) needs and assist with meeting worldwide MH workforce shortages; however, the ways to advance occupational therapy education to build occupational therapist workforce capacity in MH have been poorly addressed in the literature. This study aimed to address this knowledge gap. To our knowledge, this WFOT survey study is the first global assessment of the perspectives of occupational therapy educators regarding facilitators and barriers for enhancing MH occupational therapy education. Faculty expertise or lack thereof stood out as a facilitator or barrier across HICs and LMICs, whereas educational standards and student interest were largely perceived as facilitators. For HICs, regulation issues, lack of recognition or supervised/fieldwork practice, and lack of workforce demand were other frequently reported as barriers. In LMICs, teaching resources or applicable practice evidence stood out as additional educational barriers. All these findings are worth discussing.

Faculty expertise had large values for “high positive impact” in countries of all income levels (Figure 1), but conversely also for “high negative impact”. Taken together, these findings suggest that faculty expertise has a large positive impact when present, as well as a significant negative impact when not available or insufficient. Hence, the results indicate that larger benefits may arise from strategies to develop capacity within current or future occupational therapy faculty to deliver MH education. This comes in line with the thoughts of other scholars, as well. For example, focused on the USA as a context, a recent article by Keptner et al. [21] advocates for more mental health faculty with clinical experience in mental health. For LMICs, that need may come along with developing teaching resources and context-sensitive evidence for MH practice in low-income contexts. The direct translation of evidence from high-income countries into practice can be challenging when not adapted to the availability of human, equipment, financial, and other resources in LMICs and other low-resource contexts [35,36]. Capacity building projects [37,38] may advance the supply and MH expertise of occupational therapy educators, who, in turn, can conduct context-sensitive, practice-oriented occupational therapy MH research in LMIC contexts. Major initiatives with these goals have been undertaken for the overall MH field within LMICs [37,38,39], but we are unaware of those for occupational therapy in MH.

For HICs, larger barriers were perceived regarding the need for improved legislation and regulation, improved recognition of occupational therapy in working with people with MH needs, and improved demand for occupational therapy in MH. These findings align with HIC literature that identifies legislation and funding changes as necessary to develop or reinforce the status of occupational therapists as recognized, qualified, and reimbursed MH providers [21,40]. For example, the American Occupational Therapy Association has collaborated with state occupational therapy associations to advocate for the recognition of occupational therapists as Qualified Mental Health Providers and Qualified Behavioral Health Providers. This recognition would enable OTs to provide reimbursable mental and behavioral health services, thereby expanding access to care for individuals with mental health conditions [21]. Such strategies would likely also increase workforce demand for occupational therapy.

It should be noted that if demand increases in a context of high rates of unfilled vacancies, the few occupational therapists in practice may become overwhelmed with practice demands [41]. Increases in workforce demand must therefore be balanced with strategies to promote the attractiveness of working in MH, for example, with the provision of professional development activities, particularly for early-career MH occupational therapists [42,43,44].

HIC survey respondents identified fieldwork/supervised practice opportunities as one of the largest barriers for occupational therapy MH education. Strategies to increase MH fieldwork practice opportunities during entry-level education are therefore likely beneficial to improve the likelihood of future practice in this field [16,25,33,45]. Student interest in MH was perceived to be a large facilitator among the survey respondents, further reinforcing the value of MH fieldwork opportunities.

Education standards, such as regulatory or accreditation requirements, were rated as a large facilitator across HICs and LMICs. Of the 102 national/territorial occupational therapy associations that are members of WFOT, 88% report that occupational therapy is a regulated health profession in their jurisdiction [46]. As part of occupational therapy regulation, standards are maintained for entry-level education to ensure occupational therapists have the competencies needed for safe and effective delivery in all areas of occupational therapy practice. In addition, 74% of occupational therapy education programs across the world are approved by WFOT as meeting the *Minimum Standards for the Education of Occupational Therapists* [47]. The *Minimum Standards* require the preparation of graduates from approved occupational therapy education programs to have necessary knowledge, skills, and abilities for MH occupational therapy practice.

The results of this global survey study, alongside the related literature [25,29,30,31,32,33,34] and survey results regarding the factors affecting the occupational therapy practice in MH [24], will be used by WFOT for consultations and the stakeholder-engaged design of guidance for advancing occupational therapy in MH, in alignment with the *Global Strategy for the Occupational Therapy Workforce*. The work of WFOT, however, does not obviate the need for country-specific situational assessments and workforce planning actions, as promoted by the *Global Strategy* [14,27,48,49]. Across countries, the development of occupational therapy varies, impacting the role of occupational therapists in MH and applicable legislation and regulations [6,10,14,16,21]. Such factors can and should be considered in country-specific, regional, and local analysis and planning.

### Limitations

This survey study has a number of limitations. Although the survey was disseminated worldwide, most responses came from HICs, limiting the generalizability and analysis of results. For instance, the relatively low numbers of respondents from LMICs (*n* = 48) limited further stratification of the results into separate groups of low- and middle-income countries. The low LMIC response also reduced needed statistical power for conducting relevant, stratified inferential analysis of weighted descriptive comparisons. Nonetheless, it is worth mentioning that the rate of respondents from LMICs (31%) was more than double the rate observed in the recent global survey of occupational therapist practitioners in MH (15%) [24] and almost triple the rate received for a study examining the prevalence of occupational therapy workforce research (11%) [14,50]. Lastly, responses to the survey focused on perspectives of respondents rather than objective measures of barriers or facilitators. The results should be understood as a relative weight of factors within the context of the respondents that are compared across HICs and LMICs.

## 5. Conclusions

This paper reports on a worldwide survey of occupational therapy educators regarding factors that affect the education of occupational therapists in MH. The results identified perceived barriers and facilitators and their relative weights, stratified by the country income level of respondents (HIC and LMIC). Results indicate capacity building initiatives are required, with tailored approaches for HICs and LMICs. For instance, for LMICs, increasing the number of occupational therapy faculty with expertise in MH is likely required, along with the advancement of teaching resources. Occupational therapists might also need to conduct context-sensitive, practice-oriented occupational therapy MH research. In turn, for HICs, improved legislation/regulation and recognition of the role of occupational therapy in MH may generate improved workforce demand and supplement efforts for improving faculty capacity and supervisory/fieldwork opportunities in MH. The findings of this study, in addition to other relevant research literature, will be used by WFOT to develop global guidance for strengthening the MH occupational therapy workforce.

## Figures and Tables

**Table 1:** Survey questions and Likert-type of answers for the factors affecting occupational therapy education in mental health.

Introductory Question	Factors to be Rated as Either Facilitators or Barriers	Likert Rating Scale
Please rate the degree to which the following issues impact occupational therapy education for people with mental health needs	Regulation: Legislation/regulation regarding the role of occupational therapy in mental health	(1) “High Negative Impact” (2) “Low Negative Impact” (3) “No impact” (4) “Low Positive Impact” (5) “High Positive Impact” OR - “Unknown” - “Not applicable”
	Education standards: Education standards for occupational therapy (e.g., accreditation requirements)	
	Faculty expertise: Availability of faculty with required knowledge and experience in mental health	
	Supervised practice: Availability of fieldwork/supervised practice opportunities for occupational therapy students for working with people with mental health needs	
	Teaching resources: Availability of required teaching resources (funding, equipment, supplies)	
	Practice evidence: Availability of best practice evidence in occupational therapy mental health	
	Recognition: Recognition of the role of occupational therapy in working with people with mental health needs	
	Workforce demand: Workforce demand for occupational therapists to work with people with mental health needs	
	Student interest: Student interest in working with people with mental health needs	

## Data Availability

The datasets supporting the conclusions of this article are included within the article (and its additional files). The Supplementary File S1 contains a spreadsheet of the database used for the descriptive statistics.

## Supplementary Materials

The following supporting information can be downloaded at: https://www.mdpi.com/article/10.3390/ijerph22071009/s1, DATASET File.

## Abbreviations

The following abbreviations are used in this manuscript:

MH	Mental Health
WFOT	World Federation of Occupational Therapists

## References

[1] GBD 2019 Mental Disorders Collaborators (2022). Global, regional, and national burden of 12 mental disorders in 204 countries and territories, 1990–2019: A systematic analysis for the Global Burden of Disease Study 2019. Lancet Psychiatry.

[2] World Health Organization (2022). World Mental Health Report: Transforming Mental Health for All.

[3] Krupa T., Fossey E., Anthony W.A., Brown C., Pitts D.B. (2009). Doing daily life: How occupational therapy can inform psychiatric rehabilitation practice. Psychiatr. Rehabil. J..

[4] Galderisi S., Heinz A., Kastrup M., Beezhold J., Sartorius N. (2015). Toward a new definition of mental health. World Psychiatry.

[5] World Federation of Occupational Therapists (2019). Occupational Therapy and Human Rights (Revised).

[6] Sedgwick A., Cockburn L., Trentham B. (2007). Exploring the mental health roots of occupational therapy in Canada: A historical review of primary texts from 1925–1950. Can. J. Occup. Ther..

[7] Shepherd H.A., Jesus T.S., Nalder E., Dabbagh A., Colquhoun H. (2024). Occupational Therapy Research Publications From 2001 to 2020 in PubMed: Trends and Comparative Analysis with Physiotherapy and Rehabilitation. OTJR.

[8] García-Gestal U., Talavera-Valverde M., Souto-Gómez A.I. (2024). Occupational Therapy in Psychiatric Short-Term Hospitalization Units: Scoping Review. Community Ment. Health J..

[9] American Occupational Therapy Association (2020). Occupational Therapy Practice Framework: Domain and Process-Fourth Edition. Am. J. Occup. Ther..

[10] Read H., Zagorac S., Neumann N., Kramer I., Walker L., Thomas E. (2024). Occupational Therapy: A Potential Solution to the Behavioral Health Workforce Shortage. Psychiatr. Serv..

[11] Christie L., Inman J., Davys D., Cook P.A. (2021). A systematic review into the effectiveness of occupational therapy for improving function and participation in activities of everyday life in adults with a diagnosis of depression. J. Affect. Disord..

[12] Ikiugu M.N., Nissen R.M., Bellar C., Maassen A., Van Peursem K. (2017). Clinical Effectiveness of Occupational Therapy in Mental Health: A Meta-Analysis. Am. J. Occup. Ther..

[13] Noyes S., Lannigan E.G. (2019). Occupational Therapy Practice Guidelines for Adults Living with Serious Mental Illness.

[14] Jesus T.S., Mani K., Bhattacharjya S., Kamalakannan S., von Zweck C., Ledgerd R. (2023). Situational analysis for informing the global strengthening of the occupational therapy workforce. Int. J. Health Plan. Manag..

[15] World Federation of Occupational Therapists (2024). Top 3 Specific Practice Areas with a Shortage of Occupational Therapists. https://hr-project.wfot.org/#top3shortageAreas.

[16] Jesus T.S., Mani K., von Zweck C., Kamalakannan S., Bhattacharjya S., Ledgerd R., World Federation of Occupational Therapists (2022). Type of Findings Generated by the Occupational Therapy Workforce Research Worldwide: Scoping Review and Content Analysis. Int. J. Environ. Res. Public Health.

[17] American Occupational Therapy Association (2019). Workforce and Salary Survey Bethesda.

[18] US Bureau of Labor Statistics (2023). Occupational Employment and Wage Statistics Washinghton, DC, USA. https://www.bls.gov/oes/2022/may/naics5_621330.htm.

[19] Yan W., Ohlsen S., Wood E. (2025). Factors affecting retention of occupational therapists in adult mental health service: A systematic review with narrative synthesis. Br. J. Occup. Ther..

[20] Kaiser Family Foundation (2024). Mental Health Care Health Professional Shortage Areas (HPSAs). https://www.kff.org/other/state-indicator/mental-health-care-health-professional-shortage-areas-hpsas/?currentTimeframe=0&sortModel=%7B%22colId%22:%22Percent%20of%20Need%20Met%22,%22sort%22:%22asc%22%7D.

[21] Keptner K., Lambdin-Pattavina C., Jalaba T., Nawotniak S., Cozzolino M. (2024). Preparing for and Responding to the Current Mental Health Tsunami: Embracing Mary Reilly’s Call to Action. Am. J. Occup. Ther..

[22] The World Federation of Occupational Therapists (2019). Position Statement: Occupational Therapy and Mental Health.

[23] Jesus T.S., Landry M.D., Dussault G., Fronteira I. (2019). Classifying and Measuring Human Resources for Health and Rehabilitation: Concept Design of a Practices- and Competency-Based International Classification. Phys. Ther..

[24] Jesus T., Monteiro P., Ledgerd R., vom Zweck C. (2025). Barriers and Facilitators for the Practice of Occupational Therapy in Mental Health: Findings from a Global Practitioner Survey of the World Federation of Occupational Therapists. Preprints.

[25] Scanlan J.N., Pépin G., Haracz K., Ennals P., Webster J.S., Meredith P.J., Batten R., Bowman S., Bonassi M., Bruce R. (2015). Identifying educational priorities for occupational therapy students to prepare for mental health practice in Australia and New Zealand: Opinions of practising occupational therapists. Aust. Occup. Ther. J..

[26] Bhattacharjya S., Curtis S., Kueakomoldej S., von Zweck C., Russo G., Mani K., Kamalakannan S., Ledgerd R., Jesus T.S., World Federation of Occupational Therapists (2024). Developing a Global Strategy for strengthening the occupational therapy workforce: A two-phased mixed-methods consultation of country representatives shows the need for clarifying task-sharing strategies. Hum. Resour. Health.

[27] Jesus TSvon Zweck C., Bhattacharjya S., Mani K., Kamalakannan S., Ledgerd R. (2024). WFOT Global Strategy for the Occupational Therapy Workforce.

[28] Jesus T.S., Zweck C., Larson S., Bhattacharjya S., Kamalakannan S., Mani K., Ledgerd R. (2024). Refining the first global strategy for the occupational therapy workforce: Results from a mixed-methods survey and multimodal expert feedback. Res. Sq..

[29] Rodger S., Thomas Y., Holley S., Springfield E., Edwards A., Broadbridge J., Greber C., McBryde C., Banks R., Hawkins R. (2009). Increasing the occupational therapy mental health workforce through innovative practice education: A pilot project. Aust. Occup. Ther. J..

[30] Morley E., Rohlman D., Cheyney M., Lansing A. (2024). Impact of Training on Addressing Farmer Mental Health in Occupational Therapy Practice. OTJR.

[31] Nissen R.M., Ikiugu M.N., Barash B., Kathol M., Oorlog A. (2023). Perceptions of Occupational Therapy Educators about the Educational Preparation of Occupational Therapists for Designation as Qualified Mental Health Professionals. Occup. Ther. Health Care.

[32] Zango-Martín I., Nafai S., El Ouazzani S., Derkaoui J., Stevens-Nafai E., Codern-Bové N. (2022). Understanding the role and importance of occupational therapy in mental health services in Morocco: Perspectives from mental health professionals. Work.

[33] Scanlan J.N., Meredith P.J., Haracz K., Ennals P., Pépin G., Webster J.S., Arblaster K., Wright S., Network T.A. (2017). Mental health education in occupational therapy professional preparation programs: Alignment between clinician priorities and coverage in university curricula. Aust. Occup. Ther. J..

[34] Mahboub L., Milbourn B.T. (2015). Modernising occupational therapy teaching, research and practice in mental Health. Aust. Occup. Ther. J..

[35] Kumurenzi A., Richardson J., Thabane L., Kagwiza J., Urimubenshi G., Hamilton L., Bosch J., Jesus T. (2023). Effectiveness of interventions by non-professional community-level workers or family caregivers to improve outcomes for physical impairments or disabilities in low resource settings: Systematic review of task-sharing strategies. Hum. Resour. Health.

[36] Kumurenzi A., Jesus T.S., Richardson J., Thabane L., Kagwiza J., Cockburn L., Langhorne P., DePaul V., Melifonwu R., Hamilton L. (2025). A description of functional needs of community-dwelling stroke survivors in Rwanda: A prospective observational cohort study. Disabil. Rehabil..

[37] Endale T., Qureshi O., Ryan G.K., Esponda G.M., Verhey R., Eaton J., De Silva M., Murphy J. (2020). Barriers and drivers to capacity-building in global mental health projects. Int. J. Ment. Health Syst..

[38] Jack H.E., Merritt C., Medhin G., Musesengwa R., Mafuta C., Gibson L.J., Hanlon C., Sorsdahl K., Chibanda D., Abas M. (2020). Developing sustainable capacity-building in mental health research: Implementation outcomes of training of trainers in systematic reviewing. Glob. Health Action.

[39] Chibanda D., Abas M., Musesengwa R., Merritt C., Sorsdahl K., Mangezi W., Bandawe C., Cowan F., Araya R., Gomo E. (2020). Mental health research capacity building in sub-Saharan Africa: The African Mental Health Research Initiative. Glob. Ment. Health.

[40] Wilburn V.G., Hoss A., Pudeler M., Beukema E., Rothenbuhler C., Stoll H.B. (2021). Receiving Recognition: A Case for Occupational Therapy Practitioners as Mental and Behavioral Health Providers. Am. J. Occup. Ther..

[41] Royal College of Occupational Therapists (2023). Occupational Therapy under Pressure: Workforce Survey Findings 2022–2023.

[42] Mertala S.M., Kanste O., Keskitalo-Leskinen S., Juntunen J., Kaakinen P. (2022). Job Satisfaction among Occupational Therapy Practitioners: A Systematic Review of Quantitative Studies. Occup. Ther. Health Care.

[43] Scanlan J.N., Meredith P., Poulsen A.A. (2013). Enhancing retention of occupational therapists working in mental health: Relationships between wellbeing at work and turnover intention. Aust. Occup. Ther. J..

[44] Foster F., Palexas S., Hitch D. (2022). Early career programs for mental health occupational therapists: A survey of current practice. Aust. Occup. Ther. J..

[45] Dillon M.B., Dillon T.H., King R.M., Chamberlin J.L. (2007). Interfacing with Community Mental Health Services: Opportunities for Occupational Therapy and Level II Fieldwork Education. Occup. Ther. Health Care.

[46] World Federation of Occupational Therapists (2025). Countries/Territories Requiring National-Level Registration/Licensing. https://hr-project.wfot.org/#data_registration_required_national.

[47] World Federation of Occupational Therapists (2025). Total WFOT-Approved Education Programmes. https://hr-project.wfot.org/#data_ep_wfot_approved_total.

[48] Al Imam M.H., Jahan I., Das M.C., Muhit M., Akbar D., Badawi N., Khandaker G. (2022). Situation analysis of rehabilitation services for persons with disabilities in Bangladesh: Identifying service gaps and scopes for improvement. Disabil. Rehabil..

[49] Jesus T.S., Hoenig H. (2019). Crossing the Global Quality Chasm in Health Care: Where Does Rehabilitation Stand?. Arch. Phys. Med. Rehabil..

[50] Jesus T.S., von Zweck C., Mani K., Kamalakannan S., Bhattacharjya S., Ledgerd R. (2021). Mapping the occupational therapy workforce research worldwide: Study protocol for a scoping review. Work.

